# Perspectives on financial incentives to health service providers for increasing breast feeding and smoking quit rates during pregnancy: a mixed methods study

**DOI:** 10.1136/bmjopen-2015-008492

**Published:** 2015-11-13

**Authors:** Pat Hoddinott, Gill Thomson, Heather Morgan, Nicola Crossland, Graeme MacLennan, Fiona Dykes, Fiona Stewart, Linda Bauld, Marion K Campbell

**Affiliations:** 1 Nursing, Midwifery and Allied Health Professions Research Unit, University of Stirling, Stirling, UK; 2 Maternal and Infant Nutrition and Nurture Unit (MAINN), University of Central Lancashire, Preston, UK; 3 Health Services Research Unit, University of Aberdeen, Aberdeen, UK; 4 Department of Health Policy and Social Marketing, University of Stirling, Stirling, UK

**Keywords:** PUBLIC HEALTH, QUALITATIVE RESEARCH, PREVENTIVE MEDICINE

## Abstract

**Objective:**

To explore the acceptability, mechanisms and consequences of provider incentives for smoking cessation and breast feeding as part of the Benefits of Incentives for Breastfeeding and Smoking cessation in pregnancy (BIBS) study.

**Design:**

Cross-sectional survey and qualitative interviews.

**Setting:**

Scotland and North West England.

**Participants:**

Early years professionals: 497 survey respondents included 156 doctors; 197 health visitors/maternity staff; 144 other health staff. Qualitative interviews or focus groups were conducted with 68 pregnant/postnatal women/family members; 32 service providers; 22 experts/decision-makers; 63 conference attendees.

**Methods:**

Early years professionals were surveyed via email about the acceptability of payments to local health services for reaching smoking cessation in pregnancy and breastfeeding targets. Agreement was measured on a 5-point scale using multivariable ordered logit models. A framework approach was used to analyse free-text survey responses and qualitative data.

**Results:**

Health professional net agreement for provider incentives for smoking cessation targets was 52.9% (263/497); net disagreement was 28.6% (142/497). Health visitors/maternity staff were more likely than doctors to agree: OR 2.35 (95% CI 1.51 to 3.64; p<0.001). Net agreement for provider incentives for breastfeeding targets was 44.1% (219/497) and net disagreement was 38.6% (192/497). Agreement was more likely for women (compared with men): OR 1.81 (1.09 to 3.00; p=0.023) and health visitors/maternity staff (compared with doctors): OR 2.54 (95% CI 1.65 to 3.91; p<0.001). Key emergent themes were ‘moral tensions around acceptability’, ‘need for incentives’, ‘goals’, ‘collective or divisive action’ and ‘monitoring and proof’. While provider incentives can focus action and resources, tensions around the impact on relationships raised concerns. Pressure, burden of proof, gaming, box-ticking bureaucracies and health inequalities were counterbalances to potential benefits.

**Conclusions:**

Provider incentives are favoured by non-medical staff. Solutions which increase trust and collaboration towards shared goals, without negatively impacting on relationships or increasing bureaucracy are required.

**Strengths and limitations of this study**

- This is the first study to compare the acceptability of provider incentives for improving breast feeding and smoking cessation in pregnancy rates.
- The multidisciplinary team with extensive service-user involvement, the mixed method approach with preliminary evidence syntheses and a rigorous sampling strategy ensured diverse perspectives were included.
- The approach goes beyond existing ‘black box’ policy frameworks to understand why and how financial incentive schemes might fit within early years healthcare systems.
- The survey of early years professionals, although the largest of its kind, has potentially more limited generalisability than we hoped due to selection and response biases.
- Despite our best attempts, we failed to identify any robust strategy for UK regional or national surveys of maternity and early years health professionals due to the logistic difficulties of identifying and gaining the approval of the gatekeepers to email lists, particularly in England.

## Background

In the UK, the prevalence of smoking in pregnancy and breast feeding have shown only modest improvement in over 15 years.^1^ At the time of birth in 2010, 12% of UK women reported smoking.^1^ Breastfeeding initiation rates have shown a steady increase to over 80%, but the 55% prevalence of breast feeding at 6–8 weeks has changed little and meeting the WHO recommendation of exclusive breast feeding for the first 6 months of life seems distant.^1^ Similar issues have been reported in the USA in that while initiation rates continue to rise, continuation and exclusive breastfeeding rates remain below the *Healthy People 2020* targets.^2^ National targets for breast feeding, with up-front resources to help organisations to meet them, have not resulted in the hoped for increases in the UK. Conditional financial incentives for meeting targets, either payments or penalties, delivered to care providers at individual or system level and aligned to health policy goals are therefore attractive as a potential solution.

Kane *et al*^3^ ‘pay for prevention initiatives’ review identified several forms of provider incentives, which can broadly be categorised into two types: pay per service provided (often called fee-for-service) which may include a bonus or penalty paid based on assessed performance or a fixed payment (often called capitation or prospective payment). Provider incentive definitions are complex due to the differing health system contexts and can include a mixture of payment types, delivered at organisational, group or individual provider level. Incentivised employment contracts, like the UK government UK General Practitioner (GP) primary care Quality and Outcomes Framework (QOF) contract, can increase documentation of smoking behaviour, advice and referral rates to stop smoking services.^4^ ^5^ QOF contracts can increase protocol-driven care, resulting in greater consistency and improved organisation of care, but person-centeredness, patient satisfaction and continuity of care can decline.^6^ Some nurses report enhanced specialist skills,^6^ but little is known about the impact on provider–patient or provider–provider relationships, teamwork or morale.^4^ ^6^

Smoking cessation in pregnancy and breast feeding are potential behaviours for provider financial incentives as they have significant health, social and economic consequences.^7–11^ Systematic reviews of possible provider interventions were undertaken as part of the main Benefits of Incentives for Breastfeeding and Smoking cessation in pregnancy (BIBS) study.^12^ Our definition of a provider was: people, either individually, in groups or organisations, working in Health, Government, Voluntary Sector or other organisations, who help women to stop smoking and/or to breast feed. Our definition of incentive was purposively broad to reflect the rapid change occurring in this field and include financial (positive or negative) and non-financial tangible incentives or rewards delivered directly or indirectly at local, regional or national level. Our systematic reviews are reported in full elsewhere.^12^ They identified no provider incentive interventions to increase smoking cessation in pregnancy outcomes, one non-randomised Italian intervention of financial penalties to organisations for not meeting targets for breast feeding^13^ and two grey literature reports.^14^ ^15^ The US Joint Commission has introduced targets for exclusive breast feeding at the time of hospital discharge as one of several mandatory requirements for accreditation of maternity units with more than 1100 births per annum.^15^ A review of reviews of health service provider incentives aiming to change other healthy behaviours (weight management, exercise, alcohol and addictions), in all age groups and both sexes, was also undertaken in the BIBS study.^12^ No studies were identified where the incentive was conditional on verified patient behaviour outcomes, with most incentives conditional on provider documentation of health promotion activity. Provider incentive interventions predominantly target doctors and differences in effects of incentivising individuals or teams are uncertain.^3^ ^5^ ^6^ ^12^

In this paper, our aim was to understand the acceptability, feasibility and mechanisms of action of provider financial incentives for improving smoking cessation around childbirth and/or breastfeeding outcomes from the perspectives of early years professionals, key stakeholders and the target population of childbearing women and parents. Our definition of an early years professional is someone employed by health services in hospital or in the community whose role directly impacts on pregnant and/or postnatal women up to at least 6 months after birth. This includes doctors, midwives, health visitors, nurses, managers/administrators, allied health professionals and nursing assistants. Health visitors are qualified nurses or midwives with additional experience and training in child health, health promotion, public health and education.

## Methods

### Design

We undertook a whole systems approach to integrating the findings of the evidence syntheses described above with primary qualitative and survey research. The approach was informed by grounded theory^16^ in that there was an iterative approach to collecting data from multiple sources, analysis, refining research questions, theoretical sampling, revising interview topic guides and refining the analysis, constantly searching for disconfirming data. Service users contributed feedback throughout the study.^12^ In this paper, therefore, we report the results of surveys of health professionals and contemporaneous in-depth qualitative research.

### Study settings

The settings for the surveys of the professionals were primary and secondary early years health services across Scotland and North West England, and for the qualitative research were health, local authority, community and voluntary sector services (eg, antenatal clinics, children and family centres, mother and baby groups) in Aberdeenshire and Lancashire. Settings and participants were purposively selected for their diverse sociodemographic characteristics and their different incentive cultures for smoking cessation in pregnancy and breast feeding. Aberdeenshire had no history of incentive interventions, whereas in Lancashire incentives had recently been offered to women for smoking cessation in pregnancy^17^ and breast feeding.^18^

### Data collection for the survey of health professionals

The survey population was maternity unit staff, health visiting staff, obstetricians, paediatricians, public health specialists, GPs, practice nurses and policymakers whose work involves caring for pregnant and postnatal women and/or infants and who work in Scotland or North West England. We gained access to email lists for the population by contacting research networks, National Health Service (NHS) Research and Development (R&D) departments and Royal Colleges. In Scotland, an email with a link to the online survey was administered through:

- The Scottish Primary Care Research Network to all GP practice managers for distribution to GPs and staff involved in maternity and early years care;
- Individual R&D departments for hospital, maternity and early years staff;
- A mailing list of public health doctors;
- A mailing list of paediatricians in training;
- Two contacts at the Scottish Government for distribution to relevant maternity and early years stakeholders.

In North West England, the timing of the survey coincided with the implementation of the Health and Social Care Act on 1 April 2013. Through discussions with the Cumbria & Lancashire Research Network, and experts, it was recommended to commission Binleys (http://www.binleys.com/), a commercial organisation, to distribute the survey. The survey was sent by email in May 2013 to 4821 relevant professionals on their mailing list. Owing to a low response rate, all R&D departments within the North-West Trusts were asked to distribute the survey to relevant professionals in July 2013 and health visiting and midwifery students at University of Central Lancashire. Full details of distribution and response rates are provided elsewhere.^12^

The survey (box 1) asked about acceptability of two incentive strategies for local health service providers and were identical to two of the questions in the survey of the IPSOS MORI general public.^19^ The strategies related to (1) payments to local health services for reaching smoking cessation in pregnancy targets and (2) breastfeeding targets. Agreement with the strategies was measured on a five-point Likert scale. The questions were developed from the BIBS study evidence syntheses, service-user involvement, qualitative interviews and piloted for face validity with the target populations.^12^ ^19^ Important features of the question design arising from the developmental work was the requirement for proof from the target population that the intended behaviour had been achieved (ie, smoking cessation), due to concerns around gaming influencing the acceptability.

Box 1 **Survey questions**

Do you agree or disagree that local health services should receive additional funding if they reach targets for the number of women who prove that they have stopped smoking during pregnancy?

Precode list:

Strongly agree

Tend to agree

Neither agree nor disagree

Tend to disagree

Strongly disagree

Do you agree or disagree that local health services should receive additional funding if they reach targets for the number of women who breastfeed?

Precode list:

Strongly agree

Tend to agree

Neither agree nor disagree

Tend to disagree

Strongly disagree

We would like you to imagine that your local health service is going to run a scheme that provides incentives for stopping smoking in pregnancy. What do you think the consequence might be for participants and/or staff? (Qi) Positive consequences? (free text) (Qii) Negative consequences? (free text)

We would like you to imagine that your local health service is going to run a scheme that provides incentives for breast feeding. What do you think the consequence might be for participants and/or staff? (Qi) Positive consequences? (free text) (Qii) Negative consequences? (free text)

### Data collection for the qualitative research

A range of qualitative methods were adopted and integrated, including unstructured interviews, structured interviews with vignettes, focus groups, interactive discussions, and collaboration and feedback from service-user mother and baby groups.^12^ The purposive and theoretical sampling strategy^20^ is summarised in table 1. The initial approach was through staff working in health or community services, who gained consent for researchers to make contact with potential participants. This was flexibly implemented over time, with snowball sampling included to identify harder to reach, more disadvantaged participants and to search for disconfirming perspectives.^12^

**Table 1:** Qualitative sampling strategy

Sample	Recruitment strategy
**Pregnant women and mothers/partners/significant others** from first trimester until 6 months after birth	Researchers approached participants (through staff introduction) at pregnancy, mother and baby/toddler groups across Aberdeenshire and Lancashire Researchers approached participants (through staff introduction) at antenatal clinics, GP surgeries, hospitals, midwives across Aberdeenshire and Lancashire GPs and health visitors, midwives and voluntary workers across Aberdeenshire and Lancashire Partners/significant others through women already participating
**Providers of care/stakeholders** Midwives, health visitors, obstetricians, paediatricians, GPs, public health specialists, pharmacists, voluntary sector, children and family centre staff	Purposive or theoretical sampling: individuals identified by NHS managers, primary care networks, antenatal clinics, baby clinics. Online survey question inviting volunteers for a 15 min telephone interview/30 min face-to-face interview
**UK experts/decision-makers** UK government policymakers for maternal and child health and public health. Research ethics and research governance personnel. Expert advisers. Voluntary sector	Purposive or theoretical sampling: individuals identified through key informants and our advisory panel. Online survey question inviting volunteers for a 15 min telephone/30 min face-to-face interviewConference delegates at the: Maternal and Infant Nutrition and Nurture conference; UK National Smoking Cessation conference; Public Health in Scotland conference

GP, general practitioner; NHS, National Health Service.

An intervention vignette (box 2) of the only provider incentive strategy^13^ identified in the systematic review^12^ was used to facilitate more directed discussion. This helped the research team to gain valuable participant insights into more concrete aspects of content and delivery rather than more abstract discussion. The term ‘local health services’ was selected as the best umbrella term and qualitative data collection explored how individuals interpreted who would get the payment.

Box 2 **Intervention vignette derived from a provider incentive intervention study^13^**

The Regional Health Authority has requested local health authorities to develop local work plans and targets to increase breastfeeding rates (at birth and 16–19 weeks postnatal). All staff working within the health authority are told that a financial penalty will be applied if they do not achieve their objectives and targets.

### Survey analysis

An a priori target sample size of 1000 was set for the early years professionals survey to allow us to estimate proportions to within 3% margin of error with 95% level of confidence. A priori questions asked:

1. Is the acceptability of provider incentive strategies influenced according to sex; age (categories 18–24, 25–34, 35–44, 45–54, 55 and over); ethnicity; having children (yes, no); personal experience of smoking (never smoked, ex-smoker, current smoker—failed to stop, or no attempts to stop); had a child ever been breast fed (even if for only a day or two); job; survey region?
2. What are the independent predictors of acceptability of provider incentives?

Data were described using the appropriate summary statistics where relevant. Responses to the Likert-style outcome survey items were summarised by number, percentage and mean, and graphed using bar charts. Responses to these outcome items were tabulated, broken down by the independent predictor variables specified above. Net agreement (agree and strongly agree) and net disagreement (disagree and strongly disagree) were also reported as number and percentage. Simple and multiple ordered logit regression models were used to determine the independent predictors of acceptability for the shortlist. The relationship between predictor and outcome variables was summarised using the OR and 95% CIs. Reference categories were male; white ethnicity; doctors; no children; never smoked; child breast fed. Age was entered as 5-year categories. Job was entered as three categories: doctors; early years nursing/care staff (midwives, health visitors, maternity care staff) and other (managers, allied health professionals, researchers, support staff). All analyses were done in Stata V.13 (StataCorp, 2013, Stata Statistical Software: Release 13, College Station, Texas, USA: StataCorp LP).

### Qualitative data analysis

All qualitative data were entered into NVivo V.10 software (QSR International, Burlington, Massachusetts, USA). Analysis was informed by the framework method for applied policy research.^21^ Initially, three researchers (NC, HM, GT) identified key themes and categories independently by reading transcripts of and listening to the first four participant and four provider interviews. Through wider research team transcript reading and discussion, a single tree structure coding index was agreed. It was applied in NVivo V.10 at two sites, with 2–4 weekly merges of data sets to facilitate data organisation and retrieval to generate thematic matrices. The researchers undertook a detailed analysis of data with regular discussion several times a week between sites to ensure consistency and to search for disconfirming perspectives. Free-text responses to open questions in the health professional survey were entered onto a Microsoft Excel chart and were grouped using content analysis to triangulate the thematic qualitative data analysis. In order to focus on the variations in the acceptability, feasibility and meaning of provider incentives, separate analysis of the qualitative data was undertaken for this paper.

The collective term ‘participant’ is used within the text to indicate that all participant groups (women/partners, providers and experts) provided similar comments. When the points raised specifically refer to certain groups, this has been made explicit within the text. We refer to ‘providers’ as those who deliver a behaviour change or maintenance intervention. The qualitative findings are supported by quotations from participants followed by a reference, for example (FG1, I, providers). The first code is the participant ID number preceded by letters that relate to whether the participant took part in a focus group (FG), interactive discussion (IA), telephone interview (T), survey (S) and no letter relates to a face-to-face interview. The second code (the presence of an ‘I’) relates to whether the participant was/had been involved in an incentive programme. The last code provides a narrative description of who the participant is.

### Ethics and role of the funding source

Full National Research Ethics Service (NRES) and local ethics approval and R&D permissions were obtained (North of Scotland Research Ethics Committee (NOSRES, reference number: 12/NS/0041), University of Central Lancashire (BUSH064)).

The funders had no role in the data collection, analysis, interpretation, writing of the manuscript or the decision to submit. The qualitative research was conducted or overseen by social science and/or health researchers, three of whom had been involved in incentive interventions (GT, LB and PH). The research team included previous smokers, those with and without children, experiences of breast and formula milk feeding who held different perspectives on incentive interventions for behaviour change. Differences and potential biases were discussed in regular team meetings and noted in reflective diaries kept by the qualitative research team.

## Results

### Sample characteristics: health professional survey

There were 519 responses to the survey of health professionals. Of 519 there were 22 (4.2%) who did not answer any of the survey questions concerning the acceptability of incentive strategies, and these were excluded from all analyses. These 22 responses had extensive missing data on other survey questions and it was not possible to assess the similarity or otherwise of the excluded to included respondents. The characteristics of the 497 included respondents are shown in table 2. Midwives and GPs were the largest professional group to respond; 83% of were female and 88% were working in Scotland.

**Table 2:** Characteristics of the maternity and early years health professional sample (n=497)

Variable	Classes	Sample (%)
Sex	Male	64 (12.9)
	Female	411 (82.7)
	Missing	22 (4.4)
Age	18–34	91 (18.3)
	35–44	114 (22.9)
	45–54	182 (36.6)
	55>	85 (17.1)
	Missing	25 (5.0)
Ethnicity	White	444 (89.3)
	BME/prefer not to say	53 (10.7)
	*White British*	339 (68.2)
	*White Irish*	7 (1.4)
	*White Other*	*1 (0.2)*
	*Mixed White/Black* *Caribbean*	*1 (0.2)*
	*Mixed Other*	*1 (0.2)*
	*Asian Indian*	10 (2.1)
	*Asian Pakistani*	*2 (0.4)*
	*Asian Chinese*	*1 (0.2)*
	*Black African*	*2 (0.4)*
	*Refused*	*35 (7.0)*
Smoking status	Never smoked	370 (74.5)
	Current smoker, tried to stop smoking	17 (3.4)
	Current smoker, not tried to stop smoking	1 (0.2)
	Ex-smoker	101 (20.3)
	Declined to answer	8 (1.6)
Any children	Yes	401 (80.7)
	No	96 (19.3)
Breast feeding	Any children breast fed	387 (77.9)
	No children breast fed	110 (22.1)
Job	General practitioner	132 (26.6)
	Health visitor	47 (9.5)
	Manager	20 (4.0)
	Midwife	121 (24.4)
	Obstetrician	12 (2.4)
	Maternity staff	29 (5.8)
	Paediatrician	12 (2.4)
	Other nurse	41 (8.3)
	Public health staff	32 (6.4)
	AHP	18 (3.6)
	Support role	8 (1.6)
	Researcher	4 (0.8)
	Missing	21 (4.2)
Survey region	England	60 (12.1)
	Scotland	437 (87.9)

AHP, allied health professional; BME, black and minority ethnic.

### Sample characteristics: qualitative interviews

Interviews (55 face-to-face; 19 telephone) or focus groups (n=16) were conducted with 68 pregnant women, recent mothers and other family members; 32 service providers; 22 experts or decision-makers; and approximately 63 conference attendees. These are summarised in table 3 and the response rates to the free-text survey questions on incentive consequences are summarised in table 4. More detailed sample characteristics are provided elsewhere.^12^

**Table 3:** Qualitative study participants

Participants	Number interviewed	Totals and format
**Co-applicant mother-and-baby groups**		**Participants N=12**
Aberdeenshire	n=6	Focus groups* n=3
Blackpool	n=6	Face-to-face interviews n=2
Pregnant women and recent parents*		**Participants N=68**
Pregnant women	n=18†	Focus groups* n=8
Postnatal women	n=45	Face-to-face interviews n=19
Partners	n=5	Telephone interviews n=6
**Providers**		**Participants N=32**
Midwifery	n=8	Focus groups* n=7
Health visiting	n=12	Face-to-face interviews n=9
Doctors: paediatricians, obstetricians, GPs	n=5	Telephone interviews n=3
Public health	n=3	
Smoking cessation specialists/staff	n=2	
Voluntary sector/children's centre staff	n=2	
**Experts and decision-makers**	n=22	**Participants N=22**
		Focus groups* n=4
		Face-to-face interviews n=1
		Telephone interviews n=7
Public Health, Maternal and Infant Health Conferences	Range of participants per session involving policy, decision-makers, experts and some practitioners	**Participants N=∼63**
		Interactive recorded group discussions at conferences n=3

*A total of 16 focus groups were conducted. At three focus groups with women/recent parents a provider was present and three focus groups were a mixture of providers and experts. Two women attended two different focus groups; as did two experts (they are counted once only).

†Two pregnant women were involved in a follow-up postnatal interview (one of whom had an older child at the time of the first interview).

GP, general practitioner.

**Table 4:** Response rates to free-text questions in the professional survey (n=497 respondents)

	Positive consequences of incentives to participants and/or staff (smoking cessation)	Negative consequences of incentives to participants and/or staff (smoking cessation)	Positive consequences of incentives to participants and/or staff (breast feeding)	Negative consequences of incentives to participants and/or staff (breast feeding)
Provided comments N (%)	377 (75.9%)	372 (74.9%)	358 (72.1%)	338 (68.0%)
No data entered. N (%)	93 (18.7%)	102 (20.5%)	110 (22.1%)	121 (24.3%)
Stated ‘no consequences’ or ‘unsure’ N (%)	27 (5.4%)	23 (4.6%)	29 (5.8%)	38 (7.6%)

### Health professional survey results

#### Financial incentives for meeting smoking cessation during pregnancy targets

The responses from the health professional survey revealed net agreement with the provision of provider incentives to be 52.9% (263/497) and net disagreement was 28.6% (142/497). From a multiple ordered logistic regression model, the health visitors/maternity staff group were more likely than doctors to agree, OR 2.35 (95% CI 1.51 to 3.64; p<0.001), as were other staff, OR 2.18 (95% CI 1.38 to 3.44; p<0.001). Full details of the univariable and multivariable ordered logistic regressions are provided in web supplement 1, tables S1 and S2.

#### Financial incentives for meeting breastfeeding targets

The net agreement for incentives for meeting breastfeeding targets was 44.1% (219/417) and the net disagreement was 38.6 (192/417). From a multiple ordered logistic regression model, the predictors of agreement were:

- Health visitors/maternity care staff group were more likely than doctors to agree, OR 2.54 (95% CI 1.65 to 3.91; p<0.001), as were other staff, OR 1.94 (95% CI 1.23 to 3.05; p=0.004).
- Female respondents were more likely to agree compared with males, OR 1.79 (95% CI 1.06 to 3.91; p=0.029).
- Respondents from England compared with the reference group Scotland, OR 1.81 (1.09 to 3.00; p=0.023).

Full details of the univariable and multivariable ordered logistic regressions are provided in web supplement 1, tables S3 and S4.

In table 5, we compare the health professional agreement with the linked and separately reported British general public agreement for the same questions.^12^ Overall, more health professionals agreed with provider incentives for smoking cessation in pregnancy.

**Table 5:** Survey results comparing the acceptability of financial incentives provided to local health services for meeting targets for smoking cessation in pregnancy or breast feeding between the British general public and early years health professional

Survey sample	Payments for meeting smoking cessation targets			Payments for meeting breastfeeding targets		
	Net agreement	Neither agree not disagree	Net disagreement	Net agreement	Neither agree not disagree	Net disagreement
General public (n=1144)^19^	39.4	23.4	37.2	36.4	25	38.6
Health professionals (n=497)	52.9	18.5	28.6	44.1	17.3	38.6

In table 6, we summarise the independent predictors of agreement for the health professional responses and compare these with the linked British general public responses^19^ for provider incentives for meeting targets for proven smoking cessation in pregnancy and breast feeding. Full details of the univariable and multivariable ordered logistic regressions for the British general public survey are provided in web supplement 2, tables S5–8.

**Table 6:** Summary of the independent predictors of health professional and British general public acceptability (+) and non-acceptability (−) for provider incentives to meet targets for smoking cessation in pregnancy and breast feeding

	Survey	Age <44	Female	Non-white ethnic group	Social grade or job	Current smoker (quit attempts)	Children breast fed	Region
Local health services should receive additional funding if they reach targets for the number of women who prove that they have stopped smoking during pregnancy	British public	+++			− (Social grade C1)			– (East Midlands)− (South West; Yorkshire & Humberside; North; West Midlands; Scotland)
	Early years health professionals				+++ (midwives, health visitors/maternity staff) +++ (other staff group)			
Local health services should receive additional funding if they reach targets for the number of women who reach targets for the number of women who breastfeed	British public	++		+++				− (South West; East Midlands; Scotland)
	Early years health professionals		++		+++ (midwives, health visitors/maternity staff)+++ (other staff group)			++ (North West England)

ORs for acceptability: +, OR 1.0<1.49; ++, OR>1.50<1.99; +++, OR>2.0. Non-acceptability: −, OR>0.5<1; –, OR<0.49.

### Qualitative insights

#### Moral tensions around acceptability

Consistent with the survey results, mixed responses with regard to the acceptability of provider incentives were reported within the qualitative data. Moral tensions were evident, as providing support for health-related behaviour change and maintenance is considered integral to employment in a healthcare role, additional income or benefits for providers were seen as not warranted:

It's their job and they're getting paid for it so, no, I don't think they should get any extra for it. (24, mother)

Others recognised that incentives can motivate health professionals, who may feel demoralised for a variety of reasons, to focus on an area of health improvement as an activity with value. The UK Baby Friendly accreditation scheme^22^ can be considered an incentive scheme, as hospitals meeting quality criteria are presented with an award and plaque which is often presented in a ceremony with media coverage to mark the achievement.

At its best, it's a very positive, and very, re-enforcing of the good that you are doing, which makes people feel good about themselves on all levels, from the health care assistant right up to the manager, if it is handled properly, they feel very good about themselves, and they are incentivized to go forward because of that feeling. (IA2, I, providers & experts)

A number of the professionals considered provider incentives to be ‘insulting’ and ‘unethical’ as ‘having a [professional] relationship with a woman’ and associated increases in smoking quit and breastfeeding rates were the only incentive required. Discussion of the Cattaneo *et al*^13^ vignette (box 2) in terms of disincentives, irrespective of the fact that this intervention was effective, raised emotive responses in terms of how a ‘penalty target system’ would move away from a ‘hearts and minds’ collaboration that was needed to address these behaviours. This point was echoed, although with more negative connotations, among consumers in relation to how the ‘breast is best’ rhetoric within maternity services was more than sufficient:

I definitely wouldn't say professionals in breastfeeding [require incentives]. Because they hammer it on you enough, they don't need any incentive. They really lay it on, they spread it thick. (2, pregnant woman)

A number of the consumers, particularly those from within the more disadvantaged population groups, cited anecdotes of smoking, or not breast feeding having little impact on pregnancy, child health or development.^12^

#### Need for incentives

The need for incentives was often negatively or positively associated with resource implications, either through opportunity costs in terms of the detrimental impact on other areas of service delivery, or the substantial savings of smoking cessation and breast feeding. Examples in interviews and in the free-text responses in the survey included the prevention of ‘stillbirth‘,‘small for dates babies’, ‘lung cancer’, ‘gastroenteritis’ and other infections.

Providers often felt that the UK was making concerted efforts in rectifying the ‘appalling’ lack of postnatal care and breastfeeding support, and noted how the Baby Friendly Hospital Initiative accreditation,^22^ designed to support breast feeding and parent–infant relationships, had created ‘a massive cultural shift’ through education of the workforce, with ‘passion’, ‘motivation’ and ‘skills’ over-riding a focus on target attainment. However, as breast feeding and smoking cessation rates remain low, particularly in high deprivation areas, coupled with a lack of resources to invest in service provision, some professionals felt that they were ‘failing women’. Incentives were therefore considered a positive solution that could help organisations; ‘have it high up on their agenda’ and for additional support to be available when needed:

There's not enough hours in the day to provide the support for the women that they need. So perhaps, we do fail women in that way, so perhaps, a little bit of financial support and use this money for somebody an hour a day to come in and just help, support the women. (53, midwife)

Some participants considered that if financial incentives were shown to be financially more effective than existing strategies or where they were so concerned about the health effects, they would be willing to ‘try anything’:

My gut instinct is incentives are wrong, but as you say we've got such an issue and we have to do something and whatever we end up doing. But if you try a reward scheme, and even if it seems quite unpalatable and it works, then the justification is right there. (FG9, I, experts)

There was recognition that incentives can be divisive both between providers of care and detrimental to relationships with the women that they aim to help, particularly in the current context where services were described as ‘overstretched’. An alternative view was that incentives should be ‘for everybody’ rather than a dichotomy: either for health service providers or for women. For example, delivered to organisations, providers as well as the individual concerned to provide engagement for all involved:

I think there should definitely be some sort of target at a high level and then that should be fed down to people who are interacting with the people who you want to affect, and then if the people that have actually got to do the change, they have got to have a bit of help. (7, pregnant woman).

#### Goals of incentives

A key issue informing the decision about the acceptability of provider incentives is to define the goal to be achieved. A number of professionals argued how a focus on incentives and associated target attainment would minimise the ‘experience of the parents or their journey’. Intermediate goals, that assessed, for example, ‘enjoyment’ of breast feeding, or the extent to which parents ‘get the information at the right time’ or ‘felt supported’, were felt worthy of consideration.

The provider–woman relationship was considered crucial and concern was expressed about provider incentives for single behaviours and outcomes. A more holistic approach was suggested where the goals are decided by parents from a range of behaviours impacting on health outcomes:

Rather than just be that about stop smoking if it came from more of a health improvement function, so more about general health and what you can do to help yourself and your baby and family whilst you are pregnant. (T58, smoking awareness coordinator)

Others expressed a more negative view of targets set by organisations as dictatorial and having the potential for ‘a lot of people shouting at you [providers] to increase the breastfeeding rates’. A programme where the goal of the incentive is to reward the effort by health professionals ‘those that are doing the work and putting in the hours’ rather than targets for behaviour outcomes in women, was often considered more appropriate.

A further concern was how the goals needed to be reflective of the local community demographics. Professionals referred to how it would be ‘cruel’ and ‘unfair’ to impose unrealistic goals in areas of high deprivation where smoking and formula feeding are more prevalent, creating a situation in which providers were ‘work[ing] our guts out’ in attempts to ‘control somebody else's behaviour’ when ultimately ‘you can't make people do things that they don't want to do’:

If they told us around here that we needed 20% smoking rate and we don't get that we're penalized. We'll just go, “Oh, we're penalized now,” because there isn't any point in throwing the money into that because 10% is so far away. (FG9, experts)

Nuanced goals that were reflective of local needs were more palatable; whether this be ‘providing support for ongoing breast feeding’ in ‘middle-class’ areas, and ‘incentivising the work’ through encouraging people ‘to engage with the service’ and ‘get them to think about breast feeding in the first place’ in areas of higher deprivation. Incentive-driven goals for staff ‘to turn up to training’, for those who could demonstrate specialist knowledge, or for referrals into specialist services (for smoking cessation and/or breast feeding) particularly among professional groups who are more aligned with an incentive culture—‘evidence in GPs about changing behaviours is very convincing by giving them money for doing it’—were also highlighted.

#### Collective or divisive action

Incentive provision to all involved (consumers, providers, organisations) was considered by some participants to enable a ‘shared aim’ across different individuals and groups. Incentivising everyone in the system rather than incentivising individuals was believed to be more likely to succeed because ‘they are aiming for the same thing’. ‘Shining a light’ on practices through financial incentives and associated target setting was also perceived to be important to encourage a ‘professional approach’ and enable ‘concerted effort to change’ through specialist training to bring ‘more people up to the bar’ and dedicated service provision:

It would make sure that you've got the opportunity to make sure your staff are more highly skilled, there would be a specific focus on there or you're delivering so many hours a week delivering that service. (T51, lead health trainer)

Some professionals also considered how provider incentives could encourage individual staff members to adopt healthy behaviours, ‘do a better job’, facilitate better team work and enhance ‘job satisfaction’. However, the incongruity of incentivising a health professional to change a woman's behaviour, when the health professionals themselves chose to smoke or not breast feed was apparent.

A number of participants also considered how the ‘pressure’ of target attainment could lead to professionals being ‘manipulative’ or ‘coercive in encouraging people to participate’ with attempts focused on ‘meet[ing] that target rather than trying to support the mother’. This was believed to have possible ‘adverse effects’ on the health professional–mother relationship with potential negative implications on health behaviours:

On an individual level, that's where I get scared because if a woman felt that an individual health professional was getting some sort of payment, or incentive, or bonus or anything that persuaded her to breastfeed, it would have, I am absolutely certain it would have completely the opposite effect that you wanted. (IA2, I, providers & experts)

Some providers felt that the distinction between incentives for reaching targets versus penalties for failing to reach targets was simply a ‘linguistic difference’, while others felt strongly that penalties as discussed in relation to the Cattaneo intervention vignette (box 2) would be ‘counterproductive’ in terms of staff feeling ‘demotivated’, ‘constantly pressurised’ and create ‘low morale’.

#### Monitoring and proof

Regular monitoring as part of any provider incentive programme was considered important to provide justification for the expenditure, for accuracy in reporting and to deter gaming. However, this subject generated considerable debate due to the fallible nature of the testing methods for smoking, and problems associated with ‘proof’ of breast feeding. Carbon Monoxide (CO) monitoring was often considered an imperfect form of testing, due to levels decreasing rapidly and urine cotinine levels were considered invasive by some. Some providers felt uncomfortable counter-signing to verify breastfeeding ‘if I didn't know that it was happening all the time’ and considered that observations may be ‘too intrusive’. Furthermore, while home visits to ascertain breast feeding (evidence of formula feeding paraphernalia) as well as smoking status (house odours/ashtrays) could be undertaken, there were reservations about ‘the resources required’ and the potential for misinterpretation (eg, mother mixed feeding or another household member smoking in the home).

Concerns were also raised about ‘fraudulent’ activity among professionals due to being motivated by the ‘fear of the humiliation’ if the targets are not achieved. Regular or even ‘random’ testing was therefore considered essential by some participants to ‘prevent the study coming into disrepute’.

## Discussion

This large-scale, in-depth, mixed methods, multistakeholder study of a contentious issue demonstrated that incentives for local health service providers to meet targets for smoking cessation in pregnancy or breast feeding provoked mixed views, with health visitors and maternity staff more in favour than doctors. While there are concerns about the impact on other services, incentives might encourage investment in the skilled support services that women value, especially in the community. Outcome verification and reporting accuracy are crucial to address concerns about gaming. Many viewed targets with caution as they could potentially undermine motivation in more disadvantaged areas where staff already struggle with workload. Uniting everyone in an organisation in supporting women was recognised as a positive consequence of incentives; however, placing the responsibility unequally on one group, in this case providers of care, is potentially divisive and could result in conflict and feelings of pressure, blame and guilt. Collective and partnership approaches to concurrently incentivise women, families, communities and providers were voiced as an ideal.

This is the first study to compare the acceptability of provider incentives for breast feeding and smoking cessation in pregnancy. The approach goes beyond existing ‘black box’ frameworks^23^ to understand why and how financial incentive schemes might fit within early years healthcare systems. Strengths include the multidisciplinary team with extensive service-user involvement, the mixed methods approach with preliminary evidence syntheses^12^ and a rigorous sampling strategy to ensure diverse perspectives were included.

The survey of early years professionals, although the largest of its kind, has potentially more limited generalisability than we hoped due to selection and response biases. The response rate was lower than expected, particularly in North West England. We experienced several challenges trying to meet our a priori sample size of 1000 participants, with the result that our power to estimate responses is to within 4.4% rather than our planned 3%. The timing of the survey coincided with the reorganisation of the NHS in England in spring 2013. Survey distribution was entirely dependent on the email gatekeepers; eligible participants could have received the email from more than one source or not at all and it was not possible to calculate accurate denominators. Private companies do not appear to be the solution to accessing health professional perspectives. Despite our best attempts, we failed to identify any robust strategy for UK regional or national surveys of maternity and early years health professionals due to the logistic difficulties of identifying and gaining the approval of the gatekeepers to email lists. Discussions with academic colleagues and searching for key publications^24^ confirmed that this is a current UK challenge.

We also compared the health professional survey responses with the findings of the survey of the British general public,^19^ which may be open to criticism due to the potential selection bias for the former. However, as this research has the potential to inform important policy decisions, we juxtapose the data with a statement of caution about interpretation to provide a more complete picture. One of the reasons as to why the general public may be less likely to agree with provider incentives stems from ‘no harm’ narratives, with lack of awareness or even denial of medical evidence or resistance to being told how to behave.^12^

Health visitors and maternity care staff were more likely to agree with provider incentives and they are the professional group that such a strategy would primarily impact. The sample was predominantly women, which reflects the early years workforce and most had breast fed a child. The potential for incentives to increase resources for breast feeding and smoking cessation services appeared to be the basis for their expressed favour. Others have cautioned against attributing the failure of some incentive initiatives to self-interested and resistant health professionals.^23^ ^25^

The experience of the UK primary care QOF system, which is part of the independent contractor employment contract with the NHS, is likely to have influenced the data collected and hence generalisability to health systems in other countries. GPs have considerable freedom in how they manage practice resources. In contrast, the current QOF employment contract does not directly include early years community staff like midwives and health visitors who are employed and whose baseline salaries, unlike GPs, would not be impacted by incentive schemes. This is likely to explain some of the observed differences in acceptability between professional groups. The current context of increasing fiscal constraint, with accounts of squeezed support services around childbirth and early years and staff shortages^26^ will have impacted on our data. In our view, this strengthens the rationale for conceptualising incentives as part of complex ecological systems rather than simplistic intervention components, due to the need for public services to rapidly adapt and evolve to thrive and survive in the current fiscal climate.

Decision frameworks and checklists are available to assess when provider incentives might do more good than harm, to help prevent premature or inappropriate implementation.^27^ ^28^ Some of our themes around need, goals, accountability and monitoring map directly to these frameworks. However, they are written through a decision-maker's lens, problematise care provision in isolation and focus primarily on utilitarian values rather than a societal perspective. We argue that they are ‘jumping the gun’, as our study reveals the complexity of the crucial precursors to interventions, namely understanding their mechanisms of action and acceptability.^29^ The Medical Research Council complex intervention guidance^29^ provides the rationale for this study, which set out to build a platform for the design of incentive trials. Public patient involvement was integral and is an underpinning policy for research prioritisation and conduct in the UK.^30^ Research to date has neglected the personal perspectives and emotional responses to such incentives and the context in which they are delivered. Financial incentives provided to women contingent on breast feeding evoke concerns about the positive and negative consequences on relationships.^31^ Our ecological approach to understanding incentives as events within complex systems suggests that causal pathways are not linear and straightforward. Meanings of incentives and the context in which they are delivered is likely to impact on feasibility, effectiveness and future implementation in the real world.^32^ Others have argued that structured rigorous experimental research^33^ is needed to test different doses of incentive/penalty components of interventions, select the optimal targets for both quality improvement and achievement.^34^ Taking breast feeding as an example, financial penalties^13^ or losses of accreditation^14^ are likely to have different mechanisms of action and therefore different effect sizes from financial^15^ or accreditation gains.^22^ People are more motivated to avoid losses than they are to achieve similarly sized gains (loss aversion).^35^ Importantly, representatives of all those potentially involved: service users, families, practitioners, managers, communities, should be involved in the experimental designs to ensure an incentive systems approach with win-win goals for everyone including the tax payer. As our findings highlight, the current dichotomous conceptualisation of either patient or provider incentives may be destined to create tensions and be counter-productive. This fits the theory that individual responses to extrinsic provider incentives will be complex as health professionals have high levels of intrinsic motivation to improve patients’ health which could be crowded out with potentially detrimental consequences for healthcare.^36^ A partnership approach to intervention design^30^ should build on behaviour change theory which translates across disciplines and purposes, like SMART goal setting,^37^ monitoring and feedback^38^ and social marketing techniques like award ceremonies for achievements and dissemination of good practice. Learning from the commercial sector where incentive schemes aim to build loyalty and trust, as the process of achieving the desired behaviour, could have relevance.^39^ Equal consideration should be given to research methods which capture the unintended consequences of incentives, particularly the demotivation that can result from stigma, feelings of failure, guilt or blame.^40^

A review of pay for performance concludes that giving priority to prevention of illness would require a radical rethink of the incentives.^41^ Community commitment contracts to improve behaviours related to child health in developing countries show promise^42^ and should be a priority research area for child health innovation in developed countries. The most deprived communities where unhealthy behaviours are most prevalent could benefit; however, a counter argument is that these areas require additional funding regardless of meeting targets, as it is not an even playing field. The effect of incentivising both recipients and providers may be less than the same as or greater than the sum of the two.

## Conclusion

Our study has increased the understanding of the complexity around offering incentives to providers to change health-related behaviours for women around childbirth. Given the mixed acceptability, the lack of evidence for effectiveness and the importance of additional psychosocial support to help women to stop smoking and breast feed, experimental research is required prior to policy interventions. However, partnership and whole systems approaches are required to find a win-win incentive strategy for all stakeholders that minimises the risk of adverse consequences.
